# Performance and Usability Evaluation of an Extended Reality Platform to Monitor Patient’s Health during Surgical Procedures

**DOI:** 10.3390/s22103908

**Published:** 2022-05-21

**Authors:** Pasquale Arpaia, Egidio De Benedetto, Lucio De Paolis, Giovanni D’Errico, Nicola Donato, Luigi Duraccio

**Affiliations:** 1 Interdepartmental Research Center in Health Management and Innovation in Healthcare (CIRMIS), University of Naples Federico II, 80138 Naples, Italy; egidio.debenedetto@unina.it; 2 Augmented Reality for Health Monitoring Laboratory (ARHeMLAB), Department of Information Technology and Electrical Engineering, University of Naples Federico II, 80138 Naples, Italy; 3 Department of Engineering for Innovation, University of Salento, 73100 Lecce, Italy; lucio.depaolis@unisalento.it; 4 Department of Applied Science and Technology, Polytechnic University of Turin, 10129 Turin, Italy; giovanni.derrico@polito.it; 5 Department of Engineering, University of Messina, 98122 Messina, Italy; nicola.donato@unime.it; 6 Department of Electronics and Telecommunications, Polytechnic University of Turin, 10129 Turin, Italy; luigi.duraccio@polito.it

**Keywords:** augmented reality, extended reality, healthcare, hololens 2, surgery, real-time monitoring, Health 4.0, medical equipment, remote monitoring, uncertainty, XR

## Abstract

An extended-reality (XR) platform for real-time monitoring of patients’ health during surgical procedures is proposed. The proposed system provides real-time access to a comprehensive set of patients’ information, which are made promptly available to the surgical team in the operating room (OR). In particular, the XR platform supports the medical staff by automatically acquiring the patient’s vitals from the operating room instrumentation and displaying them in real-time directly on an XR headset. Furthermore, information regarding the patient clinical record is also shown upon request. Finally, the XR-based monitoring platform also allows displaying in XR the video stream coming directly from the endoscope. The innovative aspect of the proposed XR-based monitoring platform lies in the comprehensiveness of the available information, in its modularity and flexibility (in terms of adaption to different sources of data), ease of use, and most importantly, in a reliable communication, which are critical requirements for the healthcare field. To validate the proposed system, experimental tests were conducted using instrumentation typically available in the operating room (i.e., a respiratory ventilator, a patient monitor for intensive care, and an endoscope). The overall results showed (i) an accuracy of the data communication greater than 99 %, along with (ii) an average time response below ms, and (iii) satisfying feedback from the SUS questionnaires filled out by the physicians after intensive use.

## 1. Introduction

The Fourth Industrial Revolution has brought several benefits to different application fields, including healthcare [1]. In fact, *4.0* technologies are pervading the medical sector, such as the Internet of Things (IoT) [2,3,4], artificial intelligence [5], machine and deep learning [6,7,8], cloud computing [9], additive manufacturing [10,11,12], wearable sensors [13,14,15,16], and augmented and virtual realities (AR and VR) [17,18,19].

With regard to AR and VR, these two technologies are often referred to with an umbrella term, namely *extended reality* (XR) [20], which encompasses the entire reality-virtuality continuum and, therefore, all possible variations and compositions of real and virtual objects. In particular, relevant uses of XR in healthcare can be found in medical training [21] and surgical procedures [22,23,24,25,26,27]. An important application of XR in surgery is the overlay of digital medical images on the patients while the surgical procedure is being carried out [28]. Instead, in [29], a patient-specific hybrid simulator for orthopaedic open surgery was presented, focusing on the details for the implementation of wearable XR functionalities using Microsoft HoloLens, which has become the most successful commercial OST-HMD thanks to its advantages in contrast perception and computational effort [30]. More recently, its upgraded version (Microsoft HoloLens 2) was used to assist surgeons in completing distal interlocking [30]. Several benefits, including no radiation exposure, stereoscopic in situ visualisations, and less time consumption, were achieved with respect to conventional approaches.

Another important application of XR is the real-time monitoring of patient’s health in the operating room (OR). Patient’s vitals, along with additional information on the electronic clinical medical records, may be displayed directly on a wearable XR headset worn by the operators. The key idea is to use XR technology to allow the surgical team to effectively monitor the patient’s health status in real-time, even at a distance from the electromedical equipment. This aims to improve the efficiency of procedures by easing the burden of constantly looking at OR equipment; in this way, the surgical team can focus its attention on the patient and the task at hand, being ready to promptly act in case of aggravating conditions [31,32,33,34,35]. In [31], for example, the number of times the anaesthetist had to shift attention from the patient to the equipment was investigated. As a result, a significant decrease of more than a third through an XR head-mounted display (HMD) was observed.

Nevertheless, in the state-of-the-art, only the usability of such systems has been explored, without an assessment of their performance. The need for a usability assessment was claimed in [36], where a systematic review of 10 years of AR usability studies was provided. With regard to healthcare, it emerged that most of the medical-related AR papers were published in medical journals, and scarce qualitative data were captured from users regarding how they felt after using the system. More recently, in [37], the usability and ergonomics of the Microsoft Hololens and Meta 2 AR devices for applications in visceral surgery were investigated by using the System Usability Scale (SUS) questionnaire [38], a method successfully used for AR-based applications in education [39] and Industry 4.0 [40]. The aforementioned works, however, are strictly limited to the usability assessment. On the other hand, also the need for a performance assessment of the real-time monitoring system of patient’s undergoing surgical procedures is evident, since it is of the utmost importance to guarantee that the information is transmitted correctly and displayed in a timely manner in XR. For example, in [41,42], the key requirements regarding real-time wireless data transmission were explored, such as the transmission bandwidth, the number of interruptions per time unit, the mean duration of the stops, the monitoring delay, the energy efficiency, and the reliability. In particular, it appears that any video/audio delay greater than 300 ms should be avoided, to ensure proper interaction between the user and the system. Furthermore, fault tolerance techniques are generally included in the network to avoid network failures (which can range from small outages to large life-threatening scenarios).

Based on these considerations, in this work, an integrated monitoring platform that employs XR to assist the medical staff during surgical procedures is presented. The proposed system offers different functionalities to support the surgeon, the assistant surgeons, nurses, and anaesthetists by displaying in real-time a comprehensive set of information regarding the patient’s health status. The information to be displayed in XR can be selected by the user, based on his/her role in the procedure. The chosen XR headset (Microsoft Hololens 2) receives the data from the electromedical instruments available in the OR (e.g., respiratory ventilator, patient monitor, laparoscopic camera) and displays them in real-time [43]. Additionally, the information regarding the patient electronic clinical records is also made available upon the operator’s request. Finally, also the video streaming from the laparoscopic camera can be rendered through the platform. It should be mentioned that, in spite of the high cost of the Microsoft Hololens 2 (approximately USD 3500), its unique specifications currently makes it the most suitable device to be used to satisfy the healthcare requirements. The innovative aspect of the proposed monitoring platform lies in the comprehensiveness of the available information, in its modularity and flexibility (in terms of adaption to different sources of data), ease of use, and most importantly, a reliable communication, which are critical requirements for the healthcare field; to this aim, an evaluation of the system performance in terms of data transmission, and overall usability (by means of a SUS questionnaire), is addressed. While the obtained results in terms of data transmission outcomes and performance are crucial for medical applications, they can be readily applied also to other contexts such as for industrial or civil applications.

The paper is organised as follows. In Section 2, the concept design of the platform is presented, along with the general architecture and the communication with the adopted hardware. Section 3 describes the operation of the monitoring platform. Section 4 summarises the experimental tests carried out at the University Hospital Federico II (Naples, Italy). Finally, in Section 5, the conclusions are drawn.

## 2. Materials and Methods

This section addresses the design and implementation of the proposed XR-based monitoring platform. Particular attention is dedicated to the conceptual design of the integrated system in order to ensure modularity and flexibility (i.e., capability of connecting different medical equipment and of receiving data from different sources).

### 2.1. Design of the Monitoring Platform

**Figure 1:**
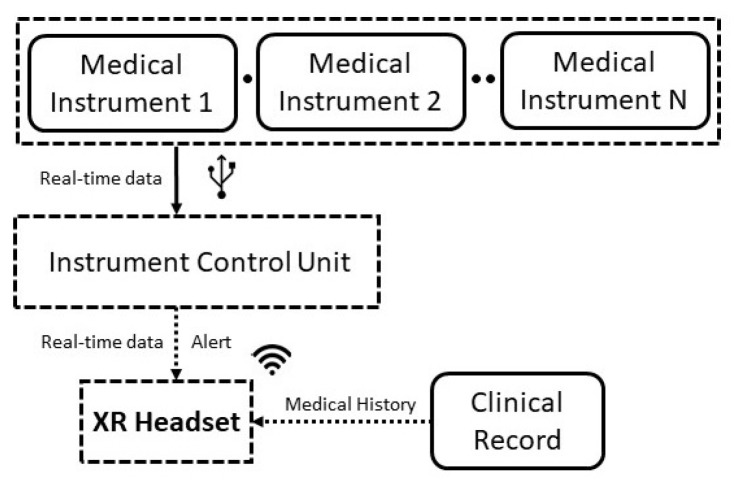
Conceptual architecture of the proposed XR-based monitoring platform.

The designed platform is conceived to help nurses, anaesthetists, and/or surgeons monitor the patients’ health status by simply wearing an XR headset. In this way, they do not have to turn to the monitoring equipment, thus being ready to promptly act in case of aggravating conditions for the patients. Figure 1 shows the different blocks of the integrated XR monitoring platform.

Basically, a set of *medical instruments* are connected via cable to an *instrument control unit (ICU)*, which sends in real-time the data to the *XR headset* worn by the user, providing, at the same time, an alert if the acquired parameters (e.g., patient’s vitals) exceed the standard values. Additionally, the XR headset receives also information about the *clinical record* of the selected patient, in order to obtain a comprehensive set of information about his/her health upon request. The design choices were made to adhere to the stringent requirements of the healthcare sector, especially in terms of communication latency between the generation and visualisation of the patient’s data.

### 2.2. Hardware

**Figure 2:**
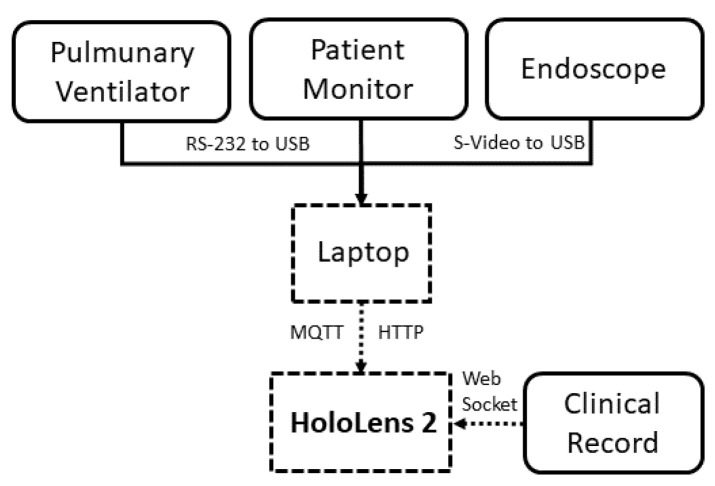
Implementation of the proposed XR-based monitoring platform.

Figure 2 shows the block diagram of the hardware and communication modalities used to implement the proposed monitoring platform. As a case study, the considered medical equipment includes instrumentation typically available in the operating room, such as: (i) a pulmonary ventilator; (ii) a patient monitor; and (iii) an endoscope for laparoscopic surgery. A laptop acts as an ICU and acquires in real-time the data from the instrumentation. Finally, it sends them to the XR headset, which, in turn, receives information on the patient from the electronic clinical record. A detailed description is provided in the following.

#### 2.2.1. Operating Room Equipment

**Figure 3:**
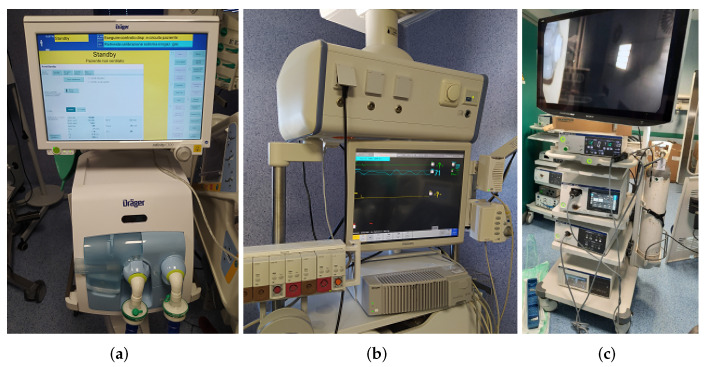
Electromedical devices used: (**a**) pulmonary ventilator; (**b**) patient monitor; (**c**) endoscope.

The OR equipment used in this work includes: (i) a pulmonary ventilator; (ii) a patient monitor; and (iii) an endoscope, as shown in Figure 3:

- *Pulmonary ventilator*: The adopted ventilator is the *Dräger Infinity V500*. It is used for intensive care and to help the lungs by administering an adequate amount of O_2_ to the patient, to eliminate the produced CO_2_, and to reduce the respiratory effort of a patient due to the excessive work of the lungs. The Infinity V500 ventilator is equipped with a local area network (LAN) interface and with three RS-232 interfaces the possibility to choose between the MEDIBUS or MEDIBUSX protocol. The baud rate, parity bits, stop bits, and terminator character can be set by the user.
- *Patient monitor*: The *Philips IntelliVue MP90* patient monitor was adopted. It allows monitoring more than 50 different vitals, such as the oxygen saturation, compound ECG, respiration rate, and heart rate, after connecting separate “plug-and-play” modules.
- *Endoscope*: The endoscope used was the *Olympus Visera Elite II*. It is an imaging platform for general surgery, urology, gynaecology, and more. It is equipped with an S-video interface, which provides access to the camera.

#### 2.2.2. XR Headset

As mentioned in Section 1, the *Microsoft HoloLens 2* was used as the XR headset. This is an OST device running *Windows 10 Holographic* and equipped with four light cameras, two infrared cameras, a depth sensor, an *inertial measurement unit (IMU)*, and an 8 MP camera. The user can interact with this device in different ways: hand gestures, eye tracking, head tracking, and voice commands. One of the most important additional features with respect to the previous version (Hololens 1) is that the diagonal field of view (FOV) is increased up to 52${}^{\circ }$. Finally, the display resolution is 2048 × 1080 pixels. In spite of the relatively high cost of this headset, its hardware and interaction modes make it the optimal solution to meet the stringent healthcare requirements, in terms of communication latency and usability.

#### 2.2.3. Laptop

The employed *operating room equipment* did not have any stringent requirements to handle the communication protocols with the instrument control unit; hence, a laptop equipped with an Intel i7-10750H processor, 16 GB RAM, Windows 10, and 3 USB 3.1 ports was chosen. It was connected to the pulmonary ventilator by means of an RS-232 to USB adapter. Instead, the connection with the patient monitor was established through (i) a *Medicollector* adapter, which is a particular LAN to RS-232 adapter, and (ii) a second RS-232 to USB adapter. Finally, the interfacing with the endoscope was realised by means of an S-video to USB adapter.

### 2.3. Software

The XR software was developed in *Unity 3D* by using the *Windows Mixed Reality Toolkit* (MRTK). A navigation menu was implemented to let the user have access in real-time to (i) the electronic clinical record and (ii) a comprehensive set of data coming from the medical equipment. Three modalities of interaction were foreseen to select the data of interest: (i) hand gestures, (ii) vocal commands, and (iii) gaze pointer. At the start of the application, the OR operator was asked to select the patient among those available. The list of patients is updated by a *WebSocket* server, which sends it to the HoloLens upon request. The XR content is provided by means of the navigation menu.

#### 2.3.1. *Navigation Menu*

**Figure 4:**
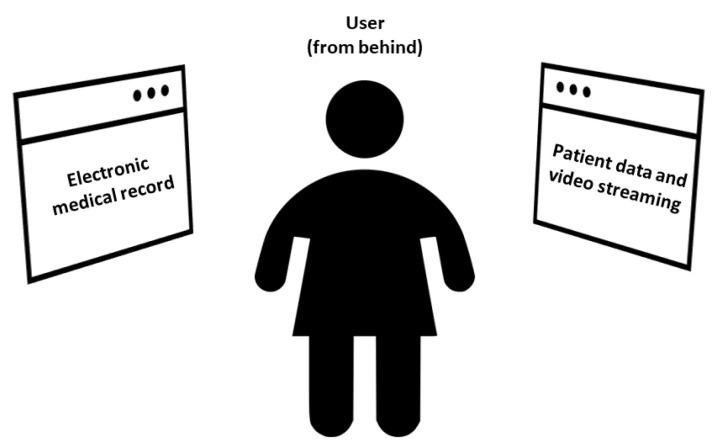
Concept of the implemented navigation menu.

The navigation menu was developed to guarantee that each window was at the same distance from the user (approximately 1 m), as shown in Figure 4. This also allows avoiding sickness effects during the use of the XR application. It consists of two main sections:

- Electronic medical record, placed originally on the left side of the menu (90° rotation of the head to the left).
- Data and video streaming from the medical equipment, placed originally on the right side of the menu (90° rotation of the head to the right).

Therefore, the view in front of the user is originally clear. However, the user can move and rotate the XR content through vocal commands, in order to show it frontally.

#### 2.3.2. *Display of Clinical Record*

The section dedicated to the display of data coming from the electronic clinical record of the selected patient is divided into four categories: (i) anamnesis, (ii) diagnostic tests, (iii) blood tests, and (iv) clinical diary. These data are sent to the HoloLens by means of the *WebSocket* protocol. In particular, the WebSocket Server provides a database of web pages for each patient. It is possible to access these web pages by means of HTTP links and a web browser. By default, Unity 3D does not foresee a browser service to display web pages in an XR environment. To this aim, the *PowerUi* asset was installed. The user can select which category to monitor by means of hand gestures or the gaze pointer.

#### 2.3.3. Interfacing with Medical Equipment: For Vital Signs and Video Streaming

With regard to the real-time interfacing with the medical equipment, HoloLens receives via *WiFi* the data coming from the laptop, which, in turn, is in charge of collecting the data from the instruments connected via cable. In particular, the laptop receives via *UART* (i) the data coming from the pulmonary ventilator, adopting the *MEDIBUS* protocol, and (ii) the video stream coming from the endoscope. Instead, the communication between the laptop and the patient monitor was implemented by means of the TCP/IP protocol via the *Medicollector* adapter. Finally, these data are sent to the HoloLens via *MQTT* (vitals) and *HTTP* (video streaming), which displays it in real-time. In this way, the OR operator can evaluate in real-time if the surgical procedure in progress is being correctly performed. Further details about the interface are provided below:

- *Acquisition from the ventilator*: A code running in the *MATLAB* environment implements the MEDIBUS protocol. This software protocol is intended to be used for exchanging data between a *Dräger* medical device and external devices via the RS-232 interface. After the initialisation of the protocol, the code asks for and decodes the vitals to be acquired. Finally, it sends them to the HoloLens via the MQTT protocol.
- *Acquisition from the patient monitor*: The code related to the data acquisition from the patient monitor was integrated with the *MATLAB* script implemented for the communication with the ventilator. This code is in charge of retrieving the waveforms from the monitor via TCP/IP through the *Medicollector* adapter. After acquiring the waveforms, the code sends them to the HoloLens via the MQTT protocol. The user can select the waveform to display by hand gestures or gaze pointer.
- *Acquisition from the endoscope*: A script running in Python 2.7 was developed to acquire the video streaming from the endoscope using the *Imutils.video* library. Successively, the data are sent in real-time to the HoloLens via the HTTP protocol.

It was chosen to adopt the HTTP protocol for the transmission of the video streaming from the endoscope to the HoloLens because the HoloLens offers native video support via the *Media Foundation* engine, which made it easy to use HTTP as a protocol for adaptive multimedia content streaming. Therefore, the *UnityWebRequest* class was used on the Unity 3D side for composing and handling HTTP requests. Instead, MQTT was adopted for the patients’ vitals’ transmission since it is a commonly used TCP-based messaging protocol for device-to-device communication, because it is lightweight (polling-free compared to RESTful over HTTP), scalable, and efficient with low-performance devices (such as low-power HMDs). The data exchanged was formatted in *JavaScript Object Notation* (JSON), a text-based format for exchanging data. On the Unity side, the *M2Mqtt* library from the *M2MqttUnity* asset was used to implement an MQTT client on the Hololens.

## 3. Operation of the XR Monitoring Platform

**Figure 5:**
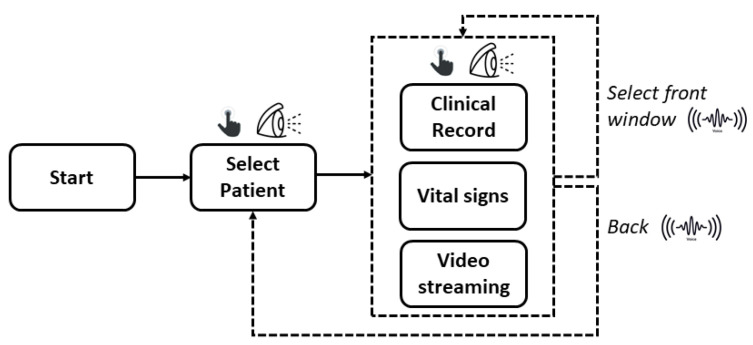
Block diagram of the user’s operation during the use of the XR platform.

Figure 5 shows the block diagram of the user’s operation while employing the XR platform.

After the user puts on the HoloLens 2 and *starts* the application, first, he/she has to *select* which *patient* to monitor. This selection can be performed by means of the gaze pointer or the hand gestures, alternatively. Therefore, the XR content appears as mentioned in Section 2.3.1. In particular, three *windows* are available:

1. *Clinical record* of the selected patient, placed originally at the left side of the navigation menu (90° rotation of the head to the left).
2. *Vital signs*.
3. *Video stream*, placed at the right side (90° rotation of the head to the right).

When the application is started, the frontal view is clear. The user can turn his/her head sideways to see the desired XR holographic content; hence, the user can select the information of interest by using the gaze pointer or the hand gestures. Alternatively, the user can also decide which window to show frontally by means of vocal commands. Finally, if he/she decides to stop the monitoring, it is possible to go back to the selection of the next patient by vocal commands.

**Figure 6:**
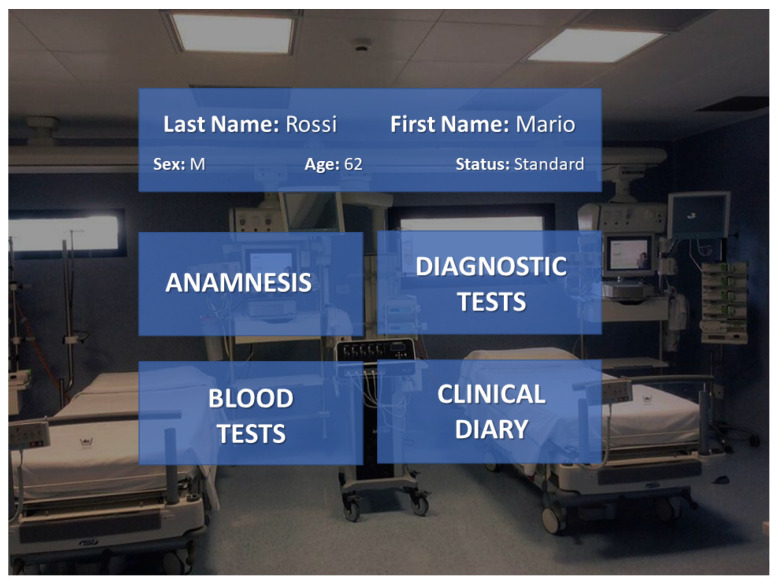
Snapshot of the electronic clinical record window.

As an example, Figure 6 shows a snapshot of what the user sees on the *Clinical Record* menu.

**Figure 7:**
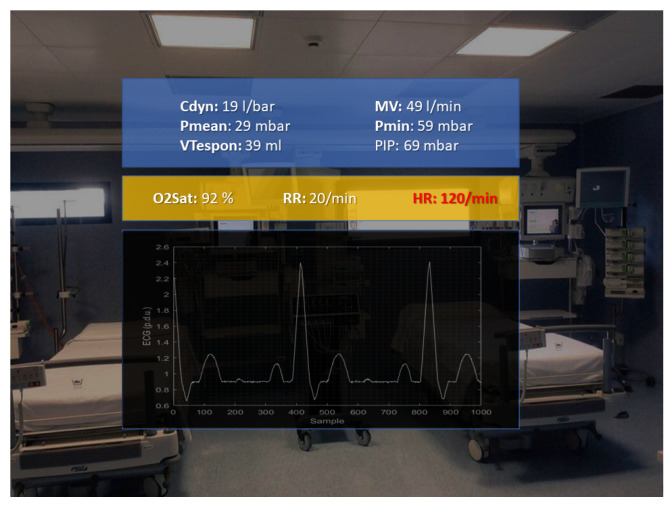
Snapshot of the vitals monitored in real-time.

On the other hand, Figure 7 shows a snapshot of the user view when he/she selects the *Vital Signs* category to monitor the patient’s vitals. In this case, the set of waveforms coming from the monitor are: heart rate, respiration rate, ECG, and O_2_ saturation. On the other hand, the monitored parameters from the ventilator are: minimum, mean, and peak airway pressure, minute volume, and compliance.

## 4. Experimental Results

After the validation of the correct functioning of the XR platform, experiments were carried out to address: (i) the real-time communication with the medical equipment; and (ii) the usability of the application running on the XR headset. To this aim, two experimental sessions were carried out, each consisting of N=5 measurement runs. A non-self-inflating bag was plugged into the pulmonary ventilator to emulate the patient’s lungs. As for the patient monitor, it was used to monitor the vitals of a healthy volunteer.

### 4.1. Performance of the Real-Time Communication

With regard to the assessment of the communication with the medical equipment, it is necessary to evaluate if the proposed integrated platform suits the stringent criteria of the healthcare field. Two figures of merit were considered: communication accuracy and time response.

The communication accuracy is defined as the percentage of packets correctly decoded by the instrument control unit. The measurement of the communication accuracy *A* was carried out for each run according to the following equation:

(1) $$A=\frac{L-E}{L}\cdot 100$$

where *L* is the number of packets sent within a run and *E* is the number of errors that occurred. Then, for each session, the accuracy mean value $\mu_{A}$ and the standard deviation $\sigma_{A}$ were assessed. Hence, the three-sigma type A uncertainty $u_{A}$ was evaluated considering the total number of runs *N*, according to the following equation:

(2) $$u_{A}=\frac{k\cdot \sigma_{A}}{\sqrt{N}}$$

with k=3, corresponding to a 99.7% confidence under the assumption of a normal distribution.

Time response *T* is defined as the time interval needed by the instrument control unit to send the data to the XR headset. For each run, the mean value $\mu_{T}i$ and the standard deviation $\sigma_{T}i$ among all the packets sent were evaluated. At the end of the session, the assessment of the weighted mean ${\bar{\mu }}_{T}$ was carried out, taking into account the different number of packets $L_{i}$ sent for each of the *N* runs, as expressed in (3).

(3) $${\bar{\mu }}_{T}=\frac{\sum_{i=1}^{N}\mu_{Ti}\cdot L_{i}}{\sum_{i=1}^{N}L_{i}}$$

With regard to the uncertainty, the three-sigma uncertainty $u_{T}$ was carried out according to the law of the propagation of uncertainties, expressed in (4).

(4) $$u_{T}=k\cdot \sqrt{\sum_{i=1}^{N}{\left(\frac{\partial {\bar{\mu }}_{T}}{\partial \mu_{Ti}}\cdot u_{Ti}\right)}^{2}}$$

where $u_{Ti}$ is the standard uncertainty of the time response evaluated for each run. Again, k=3 corresponds to a 99.7% confidence under the assumption of the normal distribution of the data. Table 1 summarises the details of the two experimental sessions. As is visible, the measured time response was below ms, while the communication accuracy assessed was greater than 99%. These obtained values are compatible with the specifications of the healthcare field.

### 4.2. System Usability

Another important aspect that was considered is the usability of the XR platform. To this aim, all the OR operators were asked to provide feedback after an intensive use of the application during the experimental trials. A modified version of the SUS questionnaire was employed, as shown in Table 2. Overall, the employed XR platform showed satisfying ergonomics (even with users with glasses and/or long hair), no motion sickness effects during the use of the application, and most importantly, ease of use. Furthermore, the multiple choice for data selection (vocal commands, gestures, gaze pointers) was particularly appreciated, thus also confirming the suitability of the Microsoft HoloLens 2 headset for the considered platform.

## 5. Conclusions

An integrated platform based on XR was proposed for the real-time monitoring of the patient’s health during surgical procedures. This platform focused on a practical use-case for members of the surgical team in the OR. Nurses, anaesthetists, or surgeons can wear an XR headset, which displays in real-time a comprehensive set of information, such as: (i) the patient electronic clinical record; (ii) the vitals acquired from a pulmonary ventilator and a monitor for intensive care; and (iii) the video stream coming from a laparoscopic camera. This monitoring platform makes the use of the data easier and available in a timely manner for the user. The proposed XR platform was developed to meet the stringent healthcare requirements, especially in terms of communication accuracy and time response. In fact, the obtained experimental results showed that the measured communication accuracy was higher than 97%, with a corresponding time response in the order of milliseconds. These values fully satisfy the aforementioned requirements of the healthcare sector. Furthermore, the usability tests through SUS questionnaires confirmed the suitability of the proposed XR monitoring platform for prolonged use. In conclusion, the proposed XR-integrated platform was demonstrated to be a suitable support for OR operators in monitoring the patients’ health during delicate surgical procedures.

## Figures and Tables

**Table 1:** Details of the two experimental sessions.

First Experimental Session				Second Experimental Session			
**L**	$\mu_{T}$ **(s)**	$\sigma_{T}$ **(s)**	**A** **(%)**	**L**	$\mu_{T}$ **(s)**	$\sigma_{T}$ **(s)**	**A** **(%)**
117	9$\;\times \;10^{-4}$	3$\;\times \;10^{-4}$	99.2	111	9$\;\times \;10^{-4}$	4$\;\times \;10^{-4}$	99.4
122	9$\;\times \;10^{-4}$	2$\;\times \;10^{-4}$	99.7	102	8$\;\times \;10^{-4}$	2$\;\times \;10^{-4}$	100.0
118	8$\;\times \;10^{-4}$	2$\;\times \;10^{-4}$	98.9	113	17$\;\times \;10^{-4}$	6$\;\times \;10^{-4}$	98.7
118	9$\;\times \;10^{-4}$	3$\;\times \;10^{-4}$	98.9	35	7$\;\times \;10^{-4}$	2$\;\times \;10^{-4}$	99.1
41	8$\;\times \;10^{-4}$	3$\;\times \;10^{-4}$	99.0	117	9$\;\times \;10^{-4}$	3$\;\times \;10^{-4}$	99.2
Total	** ${\bar{\mu }}_{T}$ **	** $u_{T}$ **	** $\mu_{A}\pm u_{A}$ **	Total	** ${\bar{\mu }}_{T}$ **	** $u_{T}$ **	** $\mu_{A}\pm u_{A}$ **
514	9$\;\times \;10^{-4}$	3$\;\times \;10^{-5}$	99.1 ± 0.4	478	11$\;\times \;10^{-4}$	6$\;\times \;10^{-5}$	99.3 ± 0.6

**Table 2:** Adopted SUS questionnaire.

N.	Question	Score				
1	I think that I would like to use this system frequently	1	2	3	4	5
2	I found the system unnecessarily complex	1	2	3	4	5
3	I thought the system was easy to use	1	2	3	4	5
4	I think that I would need the support of a technical person to be able to use this system	1	2	3	4	5
5	I think the various functions in this system were well-integrated	1	2	3	4	5
6	I thought there was too much inconsistency in this system	1	2	3	4	5
7	I would imagine that most people would learn to use this system very quickly	1	2	3	4	5
8	I found the system very cumbersome to the user	1	2	3	4	5
9	I felt very confident using the system	1	2	3	4	5
10	I needed to learn a lot of things before I could get going with this system	1	2	3	4	5
11	I found the multiple choice of data selection easy to use	1	2	3	4	5
12	I felt motion sickness effects after an intensive use of the system	1	2	3	4	5

## Data Availability

Not applicable.
